# Supplementary material for: Forecasting COVID-19-Associated Hospitalizations under Different Levels of Social Distancing in Lombardy and Emilia-Romagna, Northern Italy: Results from an Extended SEIR Compartmental Model
Source: J Clin Med. 2020 May 15;9(5):1492. doi: 10.3390/jcm9051492 (PMC7290384; doi:10.3390/jcm9051492)
Supplement: Supplementary file 1 [file jcm-09-01492-s001.pdf]

Supplementary Materials

Article title: Forecasting COVID-19-Associated Hospitalizations under Different Levels of Social Distancing in Lombardy and Emilia-Romagna, Northern Italy: Results from an Extended SEIR Compartmental Model

Journal name: Journal of Clinical Medicine

Author names: Chiara Reno, Jacopo Lenzi, Antonio Navarra, Eleonora Barelli, Davide Gori, Alessandro Lanza, Riccardo Valentini, Biao Tang and Maria Pia Fantini

Affiliation and e-mail of the corresponding author: Alma Mater Studiorum—University of Bologna, jacopo.lenzi2@unibo.it

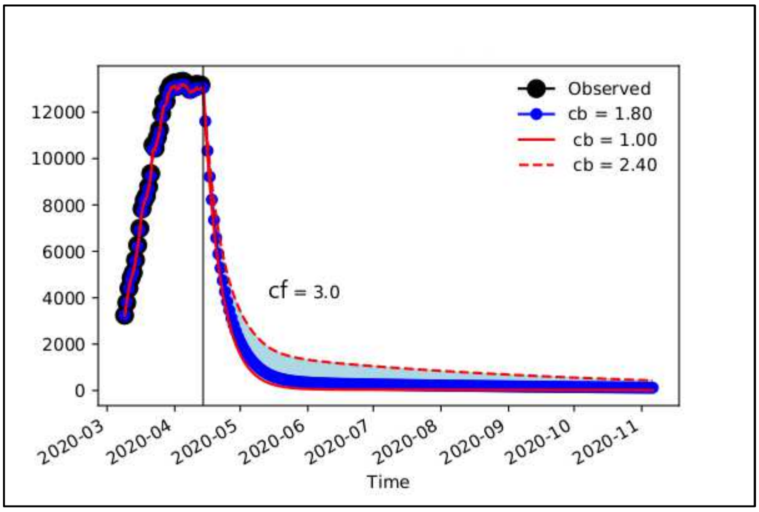

Figure S1. Lombardy's COVID-19-associated hospitalizations, assuming that on day 0 (9 March 2020) there were 5 times as many undetected infected individuals $(A + I)$ as known infected individuals $(H + L + D)$.

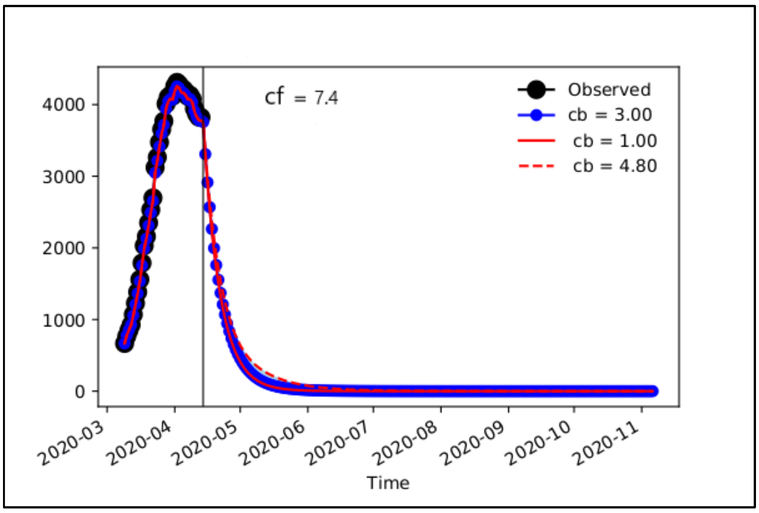

Figure S2. Emilia-Romagna's COVID-19-associated hospitalizations, assuming that on day 0 (9 March 2020) there were 5 times as many undetected infected individuals $(A + I)$ as known infected individuals $(H + L + D)$.

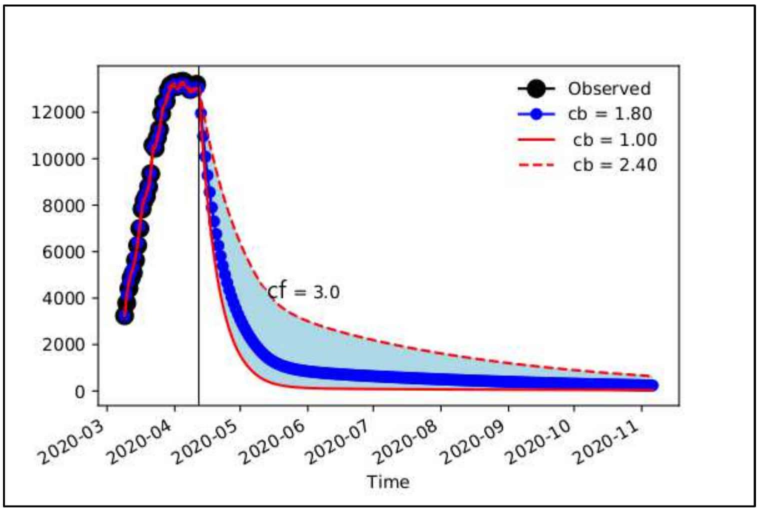

Figure S3. Lombardy's COVID-19-associated hospitalizations, assuming that on day 0 (9 March 2020) asymptomatic cases (A) were one-third of the infected pool ($A + I$).

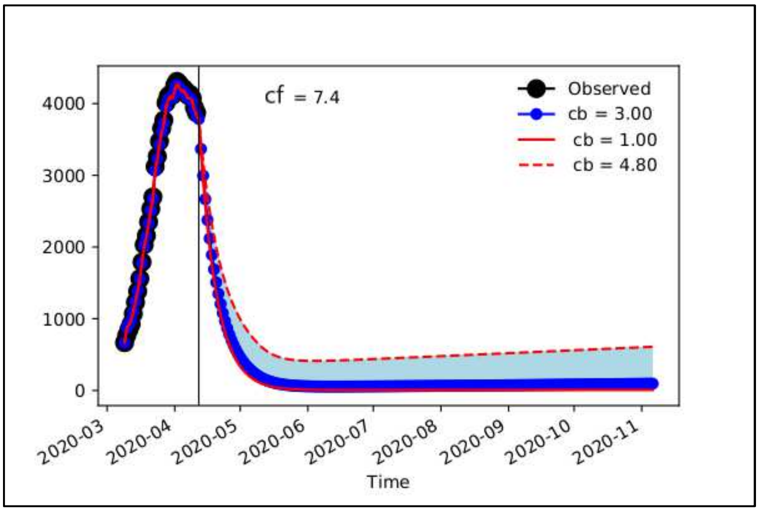

Figure S4. Emilia-Romagna's COVID-19-associated hospitalizations, assuming that on day 0 (9 March 2020) asymptomatic cases (A) were one-third of the infected pool ($A + I$).

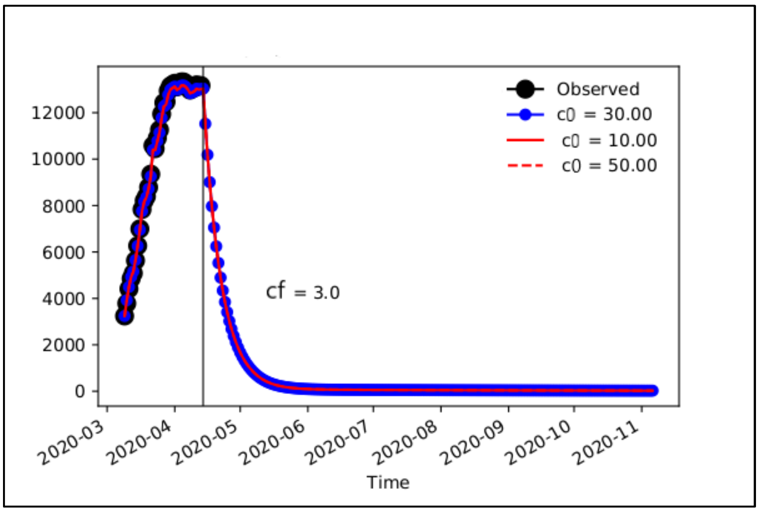

Figure S5. Lombardy's COVID-19-associated hospitalizations under different values of the contact rate on day 0 (9 March 2020).

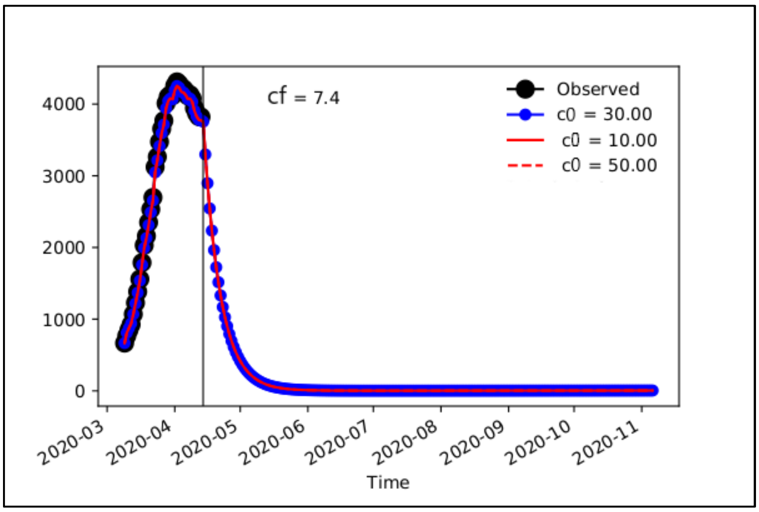

Figure S6. Emilia-Romagna's COVID-19-associated hospitalizations under different values of the contact rate on day 0 (9 March 2020).
